# Identification and validation of an immune-related gene signature predictive of overall survival in colon cancer

**DOI:** 10.18632/aging.202317

**Published:** 2020-12-19

**Authors:** Xuening Zhang, Hao Zhao, Xuezhong Shi, Xiaocan Jia, Yongli Yang

**Affiliations:** 1 Department of Epidemiology and Biostatistics, College of Public Health, Zhengzhou University, Zhengzhou 450001, Henan, China; 2 Zhengzhou University Library, Zhengzhou University, Zhengzhou 450001, Henan, China

**Keywords:** colon cancer, immune-related genes, prognostic model, tumor mutation burden, immune checkpoints

## Abstract

The heterogeneity and complexity of tumor-immune microenvironments lead to diverse immunotherapy effects among colon cancer patients. It is crucial to identify immune microenvironment-related biomarkers and construct prognostic risk models. In this study, the immune and stromal scores of 415 cases from TCGA were calculated using the ESTIMATE algorithm. AXIN2, CCL22, CLEC10A, CRIP2, RUNX3, and TRPM5 were screened and established a prognostic immune-related gene (IRG) signature using by univariate, LASSO, and multivariate Cox regression models. The predicted performance of IRG signature was external validated by GSE39582 (n=519). Stratified survival analysis showed IRG signature was an effective predictor of survival in patients with different clinical characteristics. The protein expression level of six genes was validated by immunohistochemistry analysis. Difference analysis indicated the mutation rate, immune cell of resting NK cells and regulatory T cells infiltration and four immune checkpoints of PD-1, PD-L1, LAG3 and VSIR expression levels in the high-risk group were significantly higher than those in the low-risk group. A nomogram incorporating the gene signatures and clinical factors was demonstrated had a good accuracy (1-, 3-, and 5-year AUC= 0.799, 0.791, 0.738). Our study identified a novel IRG signature, which may provide some references for the clinical precision immunotherapy of patients.

## INTRODUCTION

According to global cancer statistics, colon cancer has the fourth-highest rate of malignant tumors and the third-highest mortality rate among cancers [1]. There were approximately 1,096,601 new cases (6.1% of the total new cancer cases) and 551,269 deaths (5.8% of the total cancer deaths) from colon cancer in 2018 [2]. The traditional treatments for colon cancer are surgery, chemotherapy, and radiation. However, these treatments often have recurrent, drug-resistant, and toxic side effects, and have led to no significant improvement in colon cancer prognosis [3–5]. Immunotherapy is a new approach that has been developed in the last decade [6, 7]. In 2017, the US Food and Drug Administration first approved pembrolizumab (anti-PD-1) immunotherapy for the management of colorectal cancer [8]. Immunotherapy typically activates antitumor immunity through immune checkpoint inhibitors, such as antibodies targeting programmed cell death 1 (PD-1) and cytotoxic T-Lymphocyte antigen-4 (CTLA-4) [8]. However, due to the heterogeneity and complexity of tumor-immune microenvironments, few patients with advanced cancer have benefited from immune checkpoint inhibitors [9–11]. More and more research has focused on identifying predictive biomarkers for immunotherapy. At present, PD-L1 is the most widely accepted method. Because of the complexity of threshold quantification, PD-1 immune checkpoint cannot be used as an effective biomarker for screening advantaged populations. Therefore, it remains critical to build an effective model to accurately predict the prognosis of colon cancer patients with immunotherapy. The development of high-throughput technology and bioinformatics makes it possible to find more effective biomarkers in our big data environment.

The tumor microenvironment (TME) is generally defined as the environment surrounding the tumor, including the extracellular matrix, blood vessels, immune cells, neurons, and other cellular functions, all of which are closely related to tumor progression and therapeutic effects [12, 13]. In our research, the ESTIMATE (Estimation of Stromal and Immune cells in Malignant Tumor tissues using Expression data) algorithm was used to construct TME and identify immune-related prognostic features [14]. The response rate to immunotherapy is 52% in colon cancer patients with microsatellite instability, while immunotherapy is not effective in colon cancer patients with microsatellite stability [15]. The instability was caused by defective DNA mismatch repair mechanisms that led to somatic cell mutation, which also increased the tumor mutation burden [16]. Moreover, immune checkpoint inhibitors are only effective for specific immune-infiltrating cell subsets. For example, high tumor-infiltrating T lymphocyte content makes PD-1 and CTLA-4 monoclonal antibodies more effective. Therefore, the CIBERSORT (Cell type Identification by Estimating Relative Subpopulations of RNA Transcription) algorithm was used to analyze the relationship between the gene signature and immune cell infiltration [17].

The prognosis of colon cancer usually depends on many clinical factors, excluding the influence of genes and molecular levels. For example, the Joint American Committee on Node Metastasis’s staging of cancer has been recognized as a reference for preliminary prognostic predictions. In addition, age, sex, histological classification, and tumor location are other important factors that influence clinical outcomes and may enhance predictive value. Therefore, our study constructed a nomogram containing prognostic gene signatures and clinical prognostic factors to optimize their complementarities.

The primary aim of the present study was to investigate immune-related genes (IRG) by using the ESTIMATE algorithm to construct and validate an IRG prognostic risk score model. Second, we explored the association between IRG signature and tumor mutation burden, tumor immune cell infiltration, and immune checkpoint expression to verify the reliability of IRG and select the optimal beneficiaries of immunotherapy. Finally, a nomogram that incorporated immune-related biomarkers and clinical features was constructed to assess the immunotherapy sensitivity and prognostic characteristics for each patient.

## RESULTS

### Construction of TME, screening of DEGs and functional enrichment analysis

**Figure 1:**
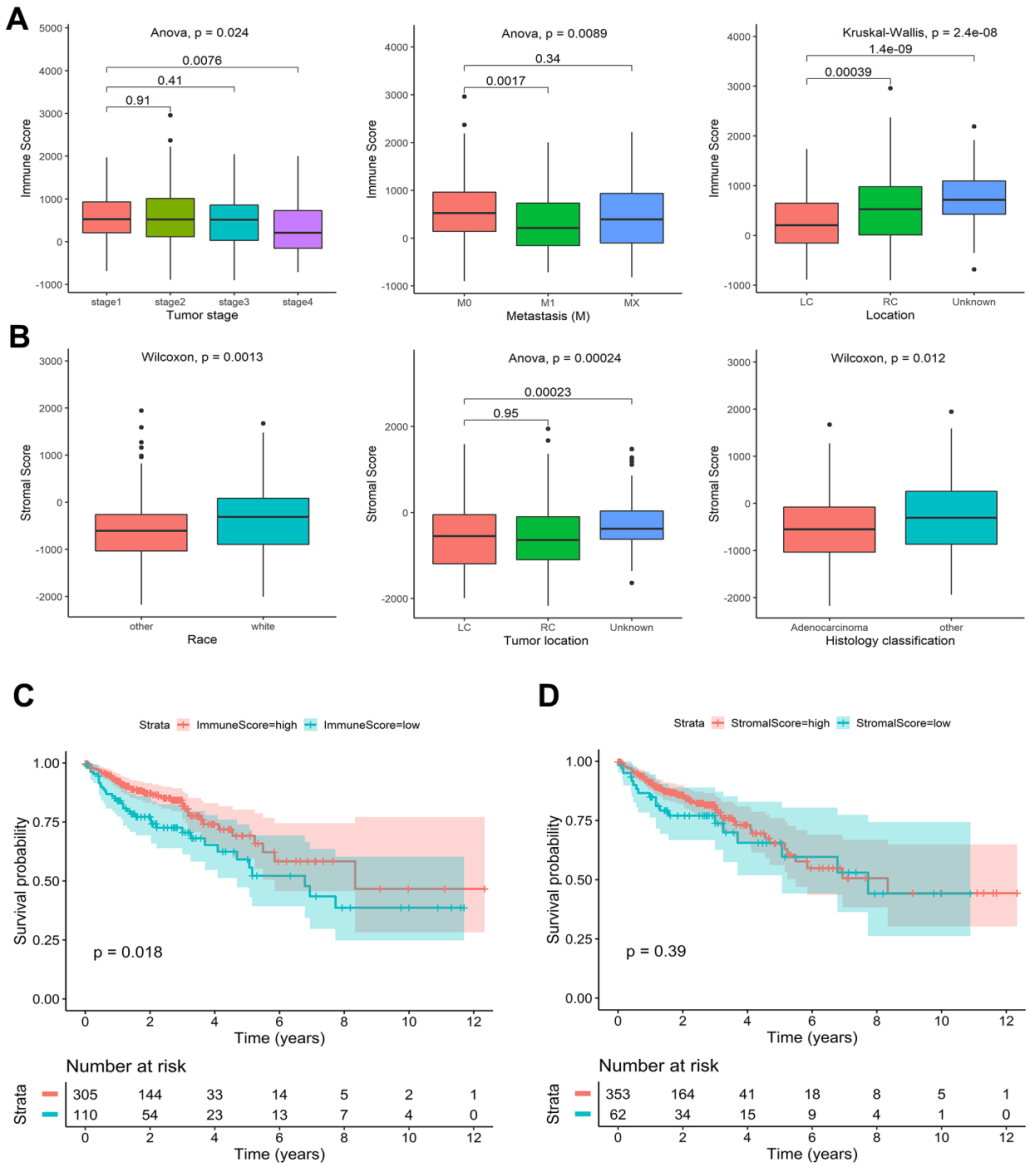
**Association of stromal and immune scores with colon cancer clinical characteristics and prognosis in TCGA.** (**A**) Significant differences in the distribution of immune scores among different tumor stage, metastasis, and tumor location groups. (**B**) Significant differences in the distribution of stromal scores among different race, tumor location, and histology classification groups. (**C**) Kaplan-Meier survival curves of high and low immune score groups. (**D**) Kaplan-Meier survival curves of high and low stromal score groups.

Detailed clinical characteristics of the training and validation cohorts are listed in Supplementary Table 1. The ESTIMATE algorithm was applied to estimate immune and stromal scores. The association of immune and stromal scores with clinical characteristics showed that the immune scores were different in pathological stage groups, tumor M staging groups, and tumor location groups. The immune score of stage I was significantly higher than stage IV (*P* = 0.008). M0 was significantly higher than M1 (*P* = 0.002), and the right side was significantly higher than the left side (*P* < 0.001) (Figure 1A). White people and non-adenocarcinomas had higher stromal scores than other races and adenocarcinomas (Race: *P* = 0.001; Histology classification: *P* = 0.012) (Figure 1B). Other variables had no significant differences in the distribution of immune scores and stromal scores (Supplementary Figure 1A, 1B). Immune scores range from -899.82 to 2959.54, and stromal scores range from -2171.21 to 1943.63. Colon cancer patients were divided into high score group and low score group by the optimal cut-off value (Supplementary Figure 1C, 1D). According to the log-rank test results, the OS of the high immune score group was significantly higher than that of the low immune score group (*P* = 0.018) (Figure 1C). There was no significant difference in OS between the high and low stromal score groups (*P* = 0.390) (Figure 1D).

**Figure 2:**
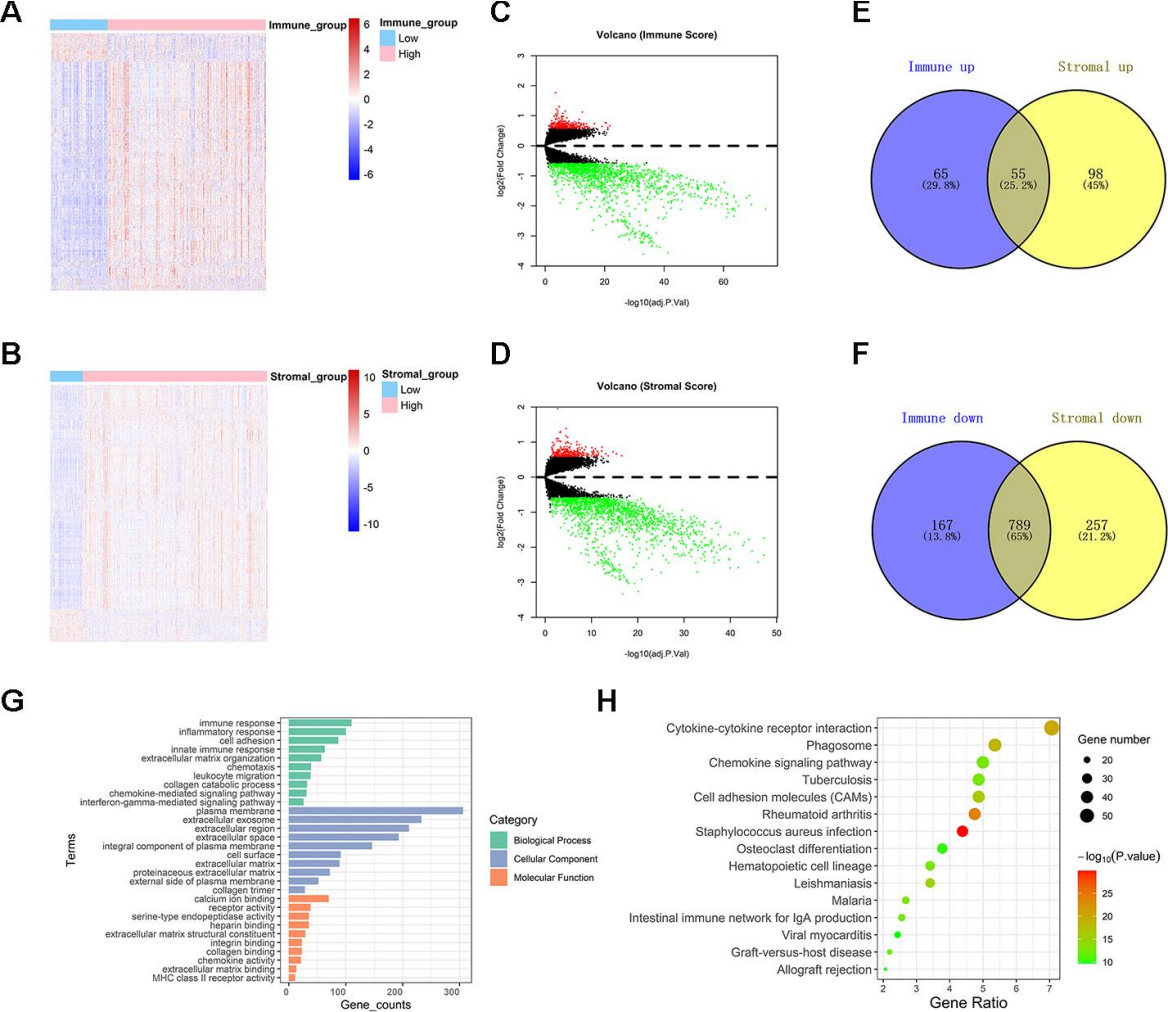
**Comparison between gene expression profiles of immune and stromal scores in TCGA.** (**A**, **B**) Heat maps showing expression profiles for immune score and stromal score-related DEGs. (**C**, **D**) Volcano plots showing up-regulated and down-regulated DEGs related to immune score and stromal score. (**E**, **F**) Venn diagrams showing the intersection of immune score and stromal score related up-regulated /down-regulated DEGs. (**G**) Histogram showing the top ten Gene Ontology terms in BP, CC, and MF. (**H**) Bubble chart exhibiting top fifteen KEGG analysis terms.

Immune- or stromal-related DEGs were identified by comparing the RNA-expression of colon cancer patients with high and low immune (or stromal) scores. A cluster analysis screened out immune-related DEGs with high score and low score groups, as displayed in the heat map (Figure 2A). A volcano plot revealed significantly differentially expressed genes (Figure 2C). A total of 1,076 genes were identified as immune-related DEGs, which contain 120 up-regulated genes and 956 down-regulated genes. The heat map and volcano plot of stromal related DEGs are shown in Figure 2B, 2D. 1,199 stromal-related genes were screened, including 153 up-regulated genes and 1,046 down-regulated genes. A Venn diagram displayed 844 intersecting immune- and stromal-related DEGs, including 55 up-regulated and 789 down-regulated genes (Figure 2E, 2F).

Top terms of GO analysis included immune response, inflammatory response, cell adhesion, and innate immune response in BP; plasma membrane, extracellular exosome, and extracellular region in CC; and calcium ion binding, receptor activity, and serine-type endopeptidase activity in MF (Figure 2G). The results of KEGG enrichment were also related to immune responses, including cytokine-cytokine receptor interaction, phagosome, chemokine signaling pathway (Figure 2H). Collectively, the results indicated that the enriched GO terms and KEGG pathways were mainly related to immune response.

### Construction, verification, and subgroup analysis of IRG risk score model

**Figure 3:**
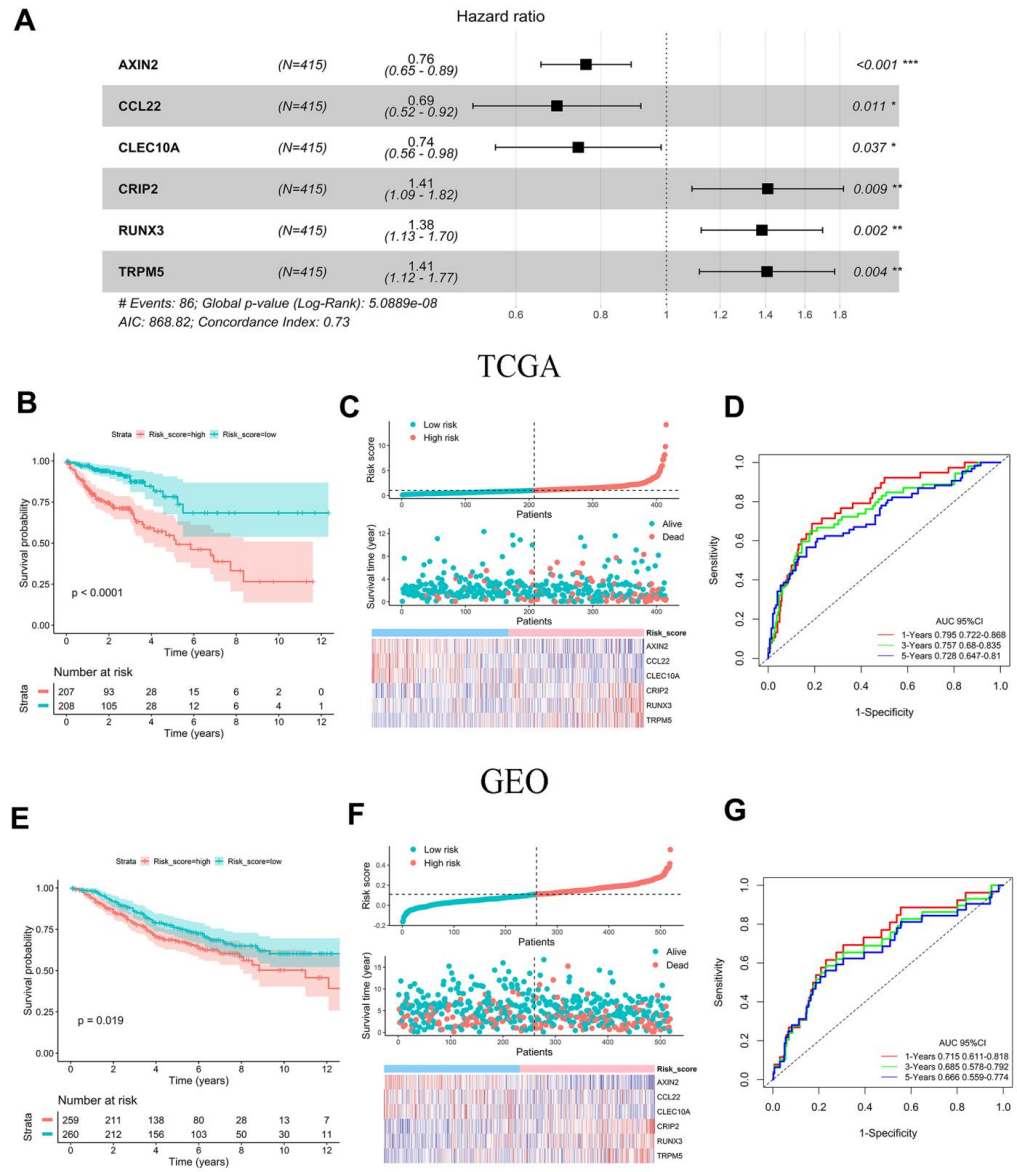
**Prognostic analysis and performance assessment of TCGA and GEO.** (**A**) Forest map showing six signature genes identified by a multivariate Cox regression analysis. (**B**, **E**) Kaplan-Meier survival curves and log-rank tests of six prognosis genes in TCGA and GEO. (**C**, **F**) Distribution of risk score, survival status and gene expression among patients in TCGA and GEO. (**D**, **G**) AUC values for 1-, 3-, and 5-year OS in TCGA and GEO.

Thirty genes related to the prognosis of colon cancer from 1096 DEGs were screened by univariate Cox analysis. The LASSO regression analysis model further identified 18 genes associated with OS (Supplementary Figure 2A, 2B). Six significant independent prognostic genes were selected by multivariate Cox regression analysis. Among them, AXIN2, CCL22, and CLEC10A were protective genes whose high expression was associated with higher survival probability. CRIP2, RUNX3, and TRPM5 were dangerous genes whose high expression was associated with a lower probability of survival (Supplementary Figure 3A, 3B). The prognostic gene risk score model = (-0.273 × expression value of AXIN2) + (-0.372 × expression value of CCL22) + (-0.299 × expression value of CLEC10A) + (0.344 × expression value of CRIP2) + (0.324 × expression value of RUNX3) + (0.341 × expression value of TRPM5) (Figure 3A). In accordance with the median risk score, all colon cancer patients from the TCGA and GEO cohorts were divided into high-risk groups and low-risk groups, respectively. The difference in OS between the two risk score groups was determined to be significant by a log-rank test (TCGA: *P* < 0.001, GEO: *P* = 0.019) (Figure 3B, 3E). The distribution of risk score, survival status, and gene expression among patients in the training set and validation set are given in Figure 3C, Figure 3F. The clustering heat maps showed that the expression of prognostic genes AXIN2, CCL22, and CLEC10A was up-regulated in the high-risk group, while the expression of CRIP2, RUNX3, and TRPM5 was down-regulated in the high-risk group (Figure 3C, 3F). The AUC for 1-, 3-, and 5-year OS were 0.795, 0.757, and 0.728 in the TCGA cohort, respectively (Figure 3D). The AUC values for 1-, 3-, and 5-year OS were 0.715, 0.685, and 0.666 in GEO cohort, respectively (Figure 3G).

**Figure 4:**
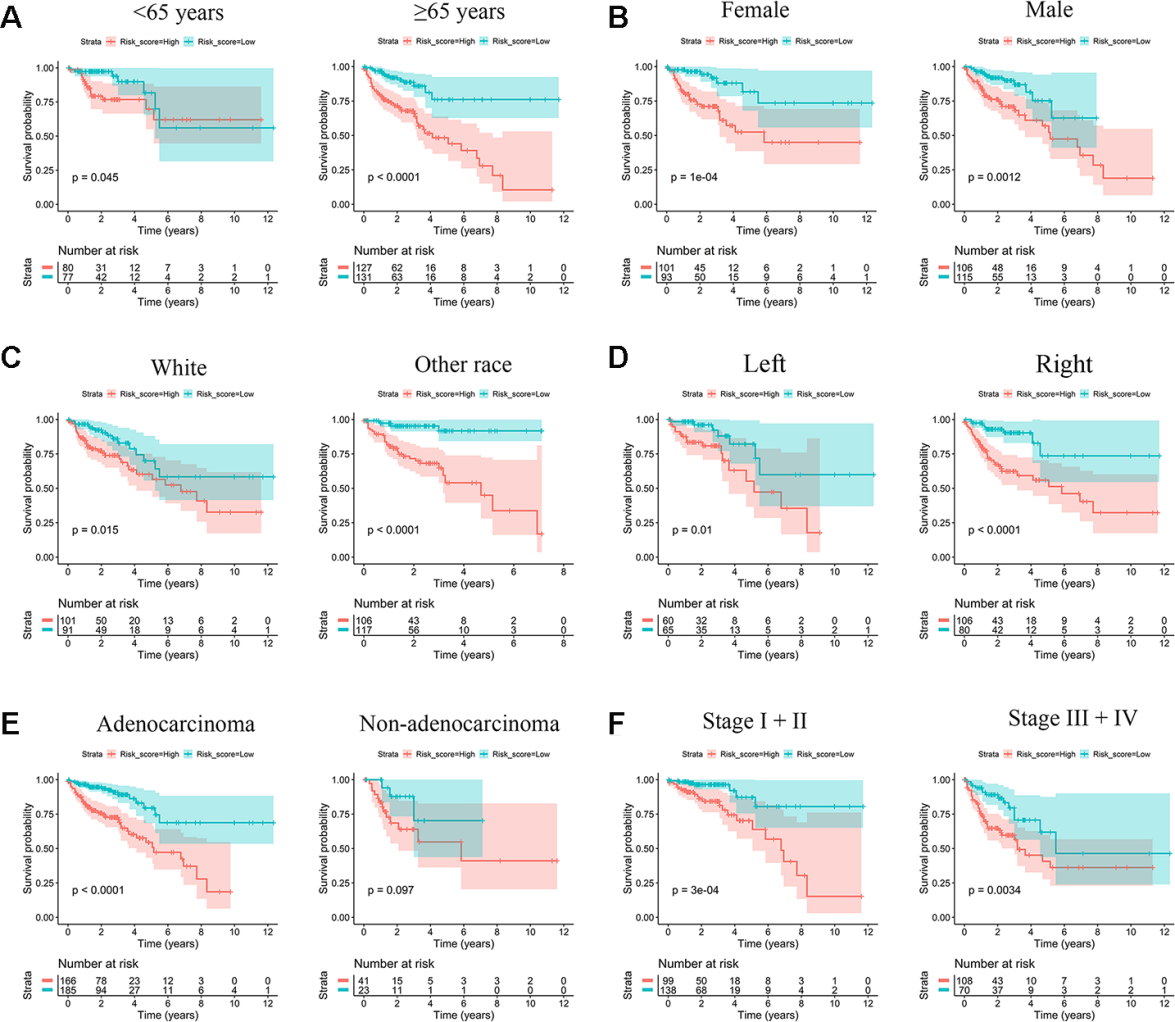
**Kaplan-Meier survival subgroup analysis according to IRG signature stratified by clinical characteristics.** (**A**) Age <65 years and age ≥65 years. (**B**) Female and Male. (**C**) White race and other race. (**D**) Left site and right site. (**E**) Adenocarcinoma and other histological type. (**F**) Stage I/II and stage III/IV.

Low-risk scores were concentrated in other races, left site, and stage I/II; all of these subgroups were associated with higher OS. However, the white race, right site, and stage III/IV had an unfavorable prognosis and accumulated significant high-risk scores (Supplementary Figure 4). Since IRG risk score is highly correlated with the above clinical characteristics, we attempted to clarify whether the IRG signature has prognostic value independent of these clinical characteristics. Stratified survival analysis showed that the log-rank tests for the survival probability of both the high- and low-risk groups were significant in different subgroups (Figure 4). IRG risk score was an effective predictor of survival in subgroups of patients with different clinical characteristics.

### Verification of expression profiles and immunohistochemistry of six immune-related prognostic genes

**Figure 5:**
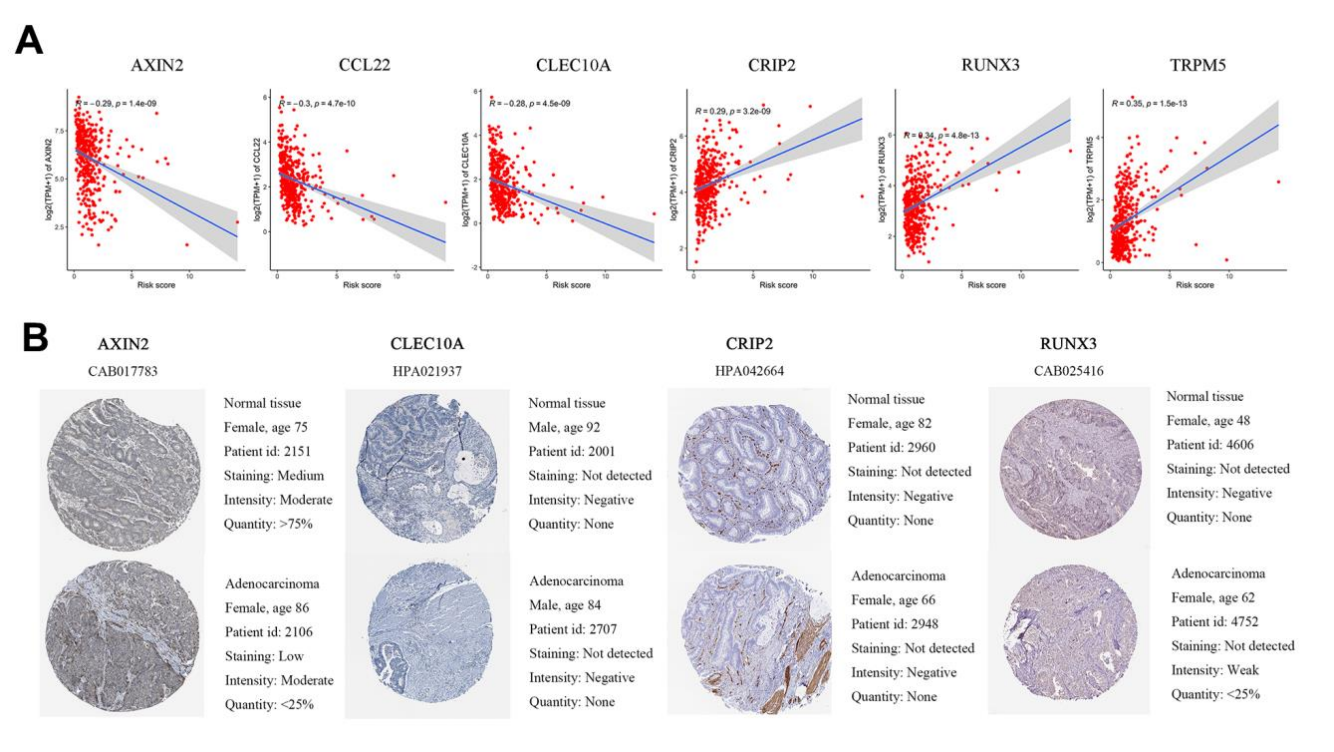
**Differences in protein expression induced by six genes were verified in human tissue samples.** (**A**) Pearson correlation of expression and risk score in GEO. (**B**) Representative immunohistochemical staining images of four genes in normal colon tissue and colon cancer specimen.

In the GSE39582 dataset, we calculated the correlation between the immune-related independent prognostic genes and risk scores. The expression levels of AXIN2, CCL22, and CLEC10A were significantly higher in the low-risk group, and the expression levels of CRIP2, RUNX3, and TRPM5 were significantly positively correlated with the risk scores (Figure 5A). This is consistent with TCGA cohort analysis.

The HPA database was used to validate the protein expression levels of six genes. CCL22 and TRPM5 were not available on the website, so we compared the other four genes’ IHC in normal tissue and tumor tissue. We found that AXIN2 staining was medium in normal tissues but low in cancer tissues, and the number of normal tissues was higher than that of cancer tissues. RUNX3 was weakly positive in tumor tissue but negative in normal tissue. CLEC10A and CRIP2 were negative in both normal and tumor tissues (Figure 5B).

### Correlations between somatic mutation, immune cell infiltration, immune checkpoints expression, and IRG signature

**Figure 6:**
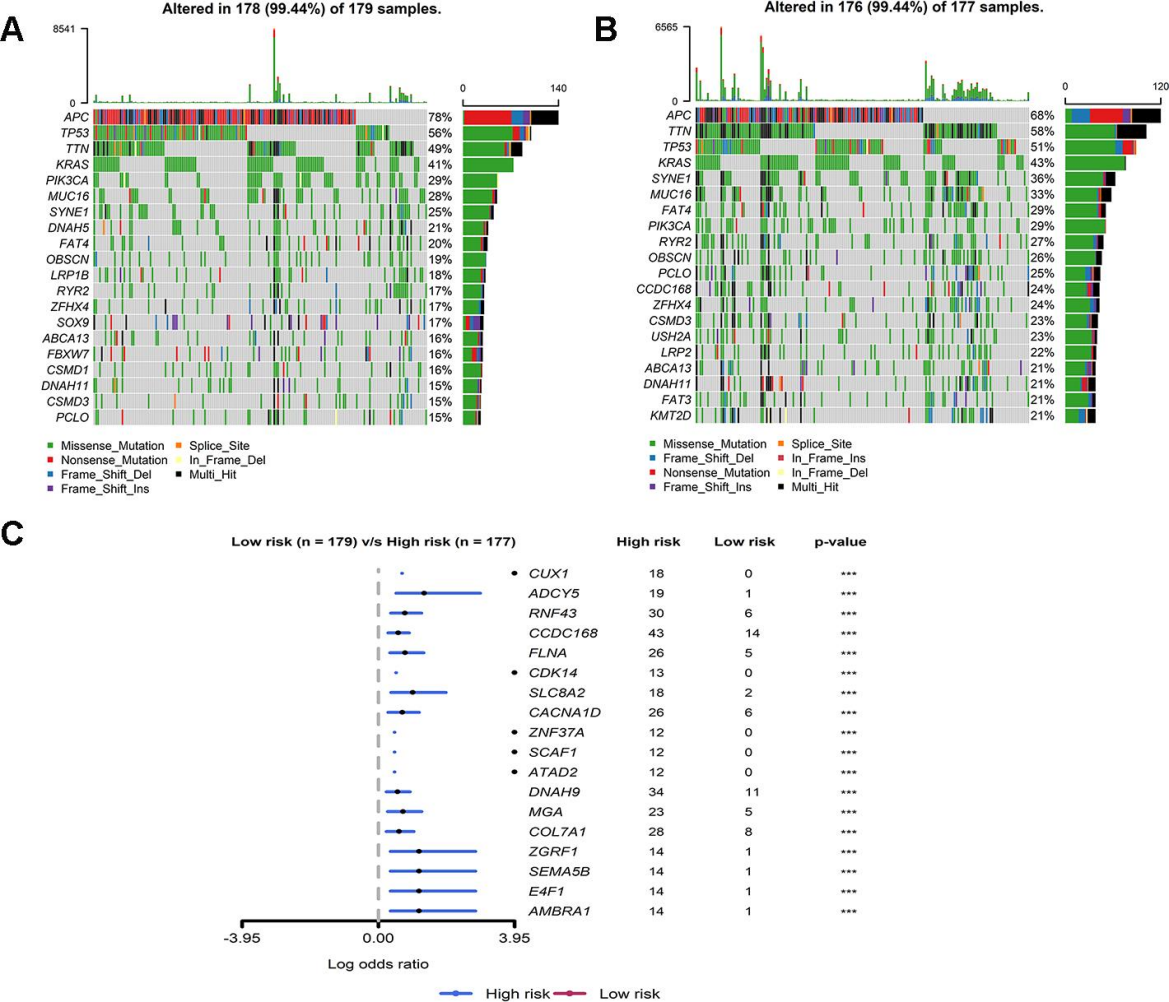
**Mutant landscape of high-risk and low-risk groups in TCGA.** (**A**, **B**) Waterfall plot representing the mutant landscape of the top 20 most frequently mutated genes in the high-risk group and low-risk group. (**C**) Forest plot representing the top 18 genes with significant differences in mutation rates between high- and low-risk groups.

By analyzing MuTect2 mutation annotation files, the total TMB and mutation distribution from the TCGA cohort (Supplementary Figure 5) were obtained. All patients with somatic mutation information were divided into either a high-risk group (n = 177) or a low-risk group (n = 179) according to the above grouping rules. The frequency of mutation of the first 20 genes in the two groups was mostly similar (Figure 6A, 6B). The two groups of mutant genes with significantly different mutation frequencies are shown in the Figure 6C. The frequencies of all mutated genes were higher in the high-risk group than in the low-risk group, indicating that the frequency of somatic mutation was positively correlated with our IRG risk score (Figure 6C). The relationship between the IRG risk score and the first eight mutation pathways is shown in Supplementary Table 2. The mutation rate of RTK-RAS, NOTCH, MYC, Cell-Cycle, and TCF-Beta pathways in the high-risk group was significantly higher than that in the low-risk group. Taken together, these results suggested that colon cancer tumor cells in the high- and low-risk groups may have different mutation driver genes and pathways.

**Figure 7:**
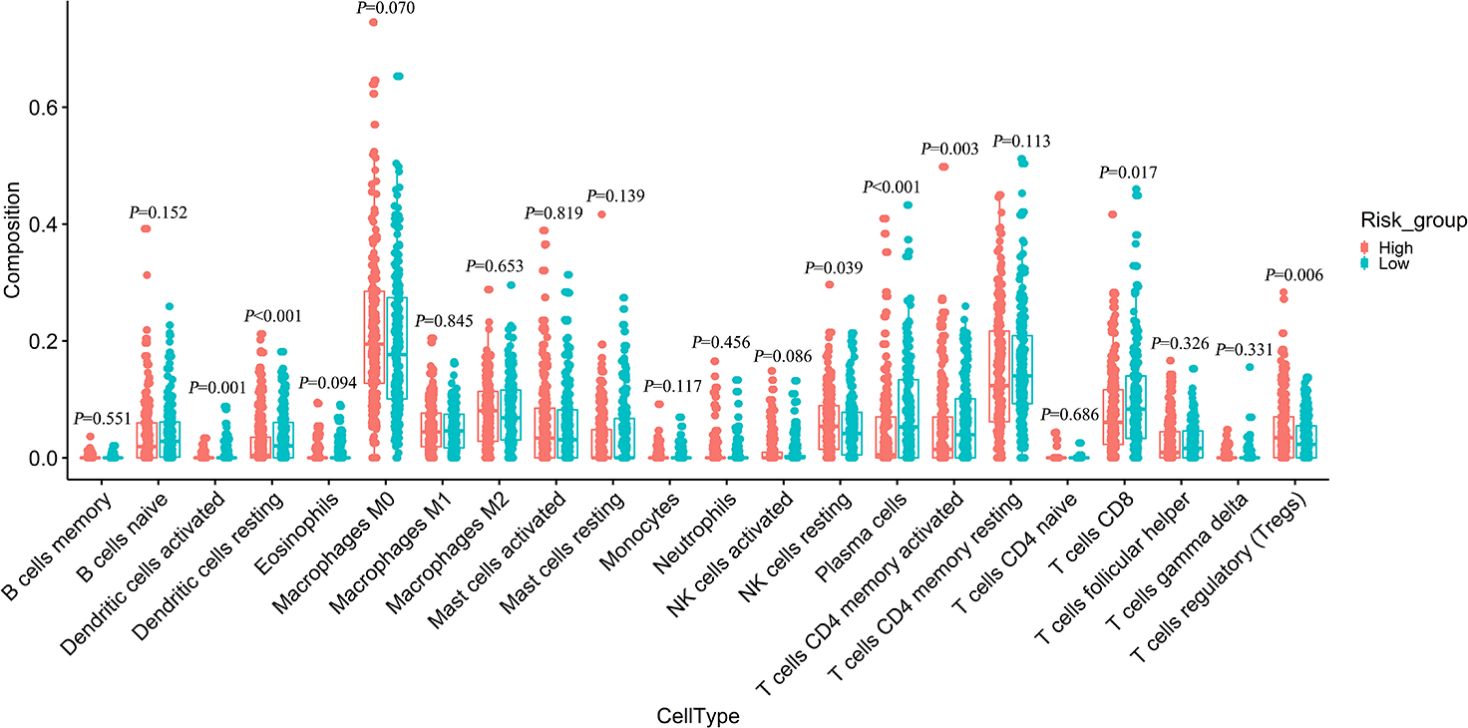
**Difference analysis of 22 immune cells infiltration between high- and low-risk groups.**

The “CIBERSORT” algorithm was used to estimate the difference in immune infiltration between low-risk and high-risk colon tumors in 22 immune cell subsets. The composition of immune cells in the high-risk and low-risk samples is shown in Supplementary Figure 6A. The difference in the proportion of immune infiltrating cells between the high-risk and low-risk samples was exhibited on the heat map (Supplementary Figure 6B). The immune cells with significantly higher infiltration in the high-risk samples were resting NK cells, and regulatory T cells (*P* < 0.05). The infiltration of CD8 T cells, plasma cells, memory-activated CD4 T cells, resting dendritic cells, and activated dendritic cells were significantly higher in the low-risk samples than in the high risk samples (*P* < 0.05) (Figure 7). Therefore, different immune infiltrators in colon cancer patients might be used as prognostic indicators and targets of immunotherapy.

**Figure 8:**
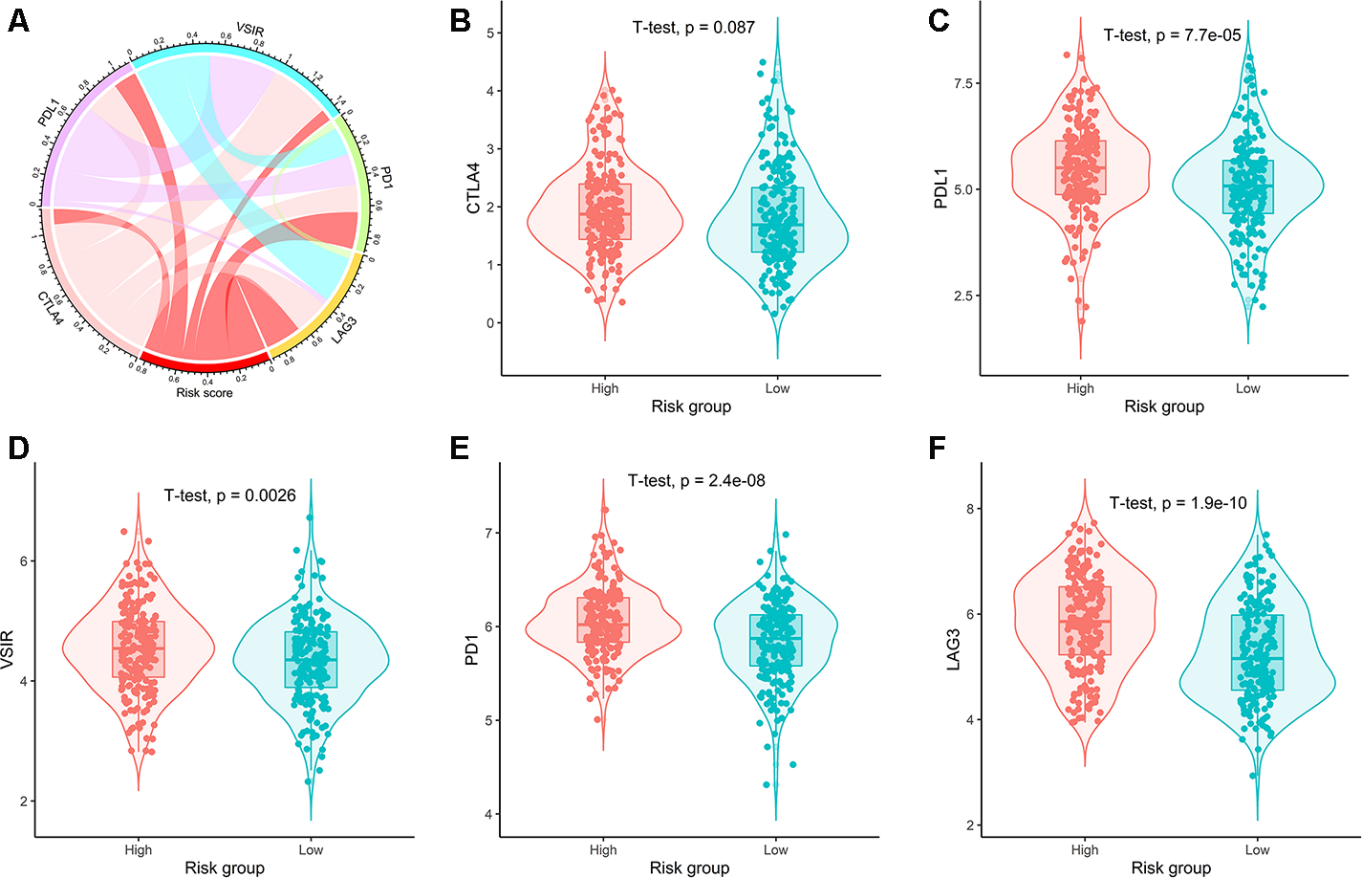
**Correlation of risk scores with expression of five prominent immune checkpoints.** (**A**) Circular plot visualizing correlation coefficient of risk scores with expression of five common immune checkpoints. Box plots showing comparison of the expression of (**B**) CTLA4, (**C**) PDL1, (**D**) VSIR, (**E**) PD1, and (**F**) LAG3 between high- and low-risk groups.

The correlation between the risk scores and expression of five common immune checkpoints was shown on the circular plot (Figure 8A). The results showed that the risk scores were significantly positively correlated with the expression of PD-1, PD-L1, LAG3, and VSIR. The expression of CTLA-4 in high and low risk groups had no significant difference (Figure 8B). The expression of PD-1, PD-L1, LAG3 and VSIR in the high-risk group was significantly higher than that in the low-risk group (Figure 8C–8F).

### Independent prognostic validation of IRG risk score and construction of a nomogram

**Figure 9:**
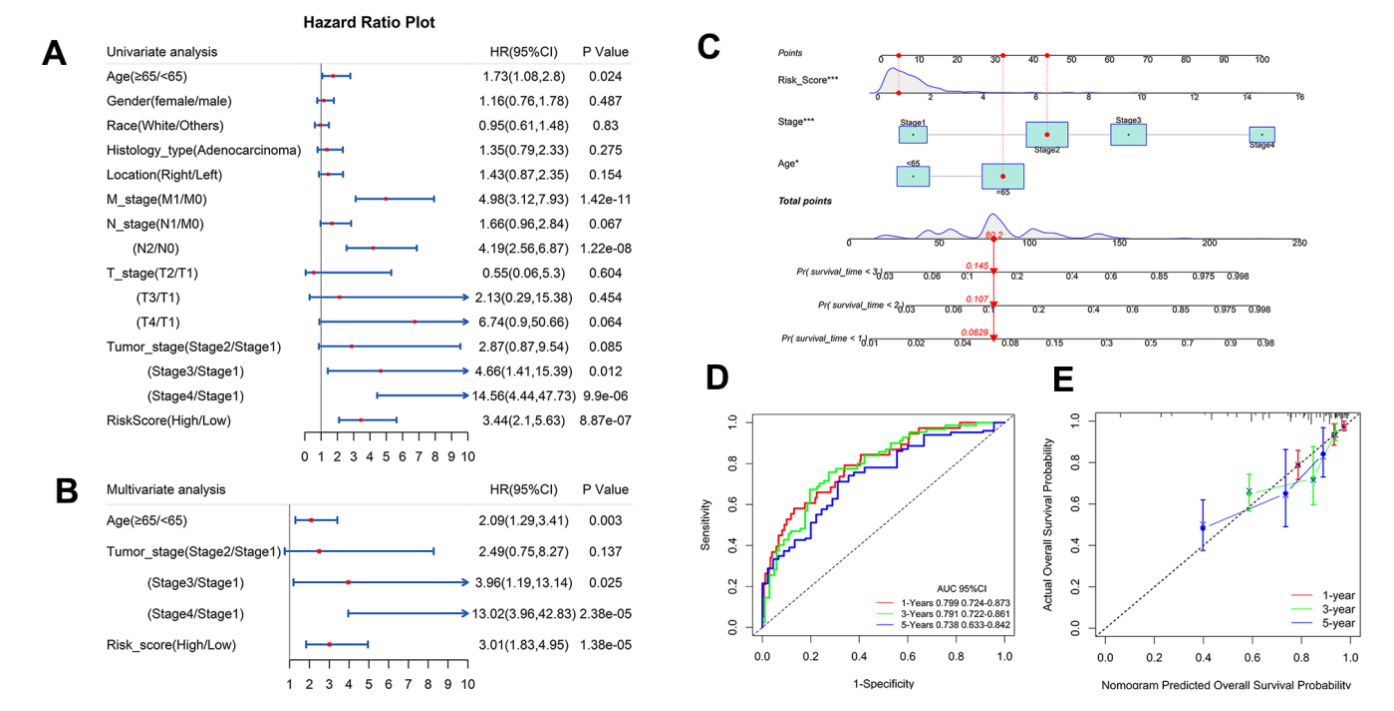
**Independent analysis of IRG and construction of nomogram and performance assessment.** (**A**, **B**) Univariate and multivariate Cox regression analysis of prognostic factors in TCGA. (**C**) Nomogram based on clinical factors and risk grads in TCGA. (**D**) AUC values for 1-, 3-, and 5-year survival rates in a nomogram. (**E**) Calibration for the possibility of 1-, 3-, and 5-year survival in a nomogram.

To explore whether IRG risk score is an independent predictor of colon cancer, age, gender, histological classification, pathological stage, tumor invasion, lymph node, metastasis, and tumor location were incorporated in the univariate analysis. The analysis indicated that age, metastasis, lymph node, tumor stage, and risk score were related to prognosis (Figure 9A). Risk score stratified and the meaningful clinical factors selected by univariate analysis were combined into a multivariate Cox regression analysis. There was collinearity among metastasis, lymph node, and tumor invasion and tumor stage. The multivariate Cox regression model did not include meaningful factors such as metastasis and lymph node. Old age (≥ 65 years: HR 2.09; 95% CI, 1.29–3.41), high pathological stage (stage III: HR 3.96; 95% CI, 1.19–13.14; stage IV: HR 13.02; 95% CI, 3.96–42.83), and high-risk score (HR 3.01; 95% CI, 1.83–4.95) were independent prognostic factors (Figure 9B).

A nomogram including risk score, age, and pathological stage was constructed that could visualize prognostic risk factors and provide a quantitative method for predicting the survival probability of colon cancer patients (Figure 9C). The AUC values of the nomogram for 1-, 3-, and 5-years were 0.799, 0.791, and 0.738 respectively (Figure 9D). The calibration of the nomogram for the possibility of 1-, 3-, and 5-year survival suggested strong coherence between the prediction and actual observations (Figure 9E).

### Comparison of the IRG signature using the ESTIMATE algorithm with models using other methods

To determine whether the immune-related gene signature obtained with the ESTIMATE algorithm was superior to other models, our model was used to compare it with the CIBERSORT and IMMPORT algorithms. Nine immune-related gene signature based on the CIBERSORT algorithm showed that the AUC values of 3-year and 5-year OS were 0.676 and 0.661, respectively. Five immune-related genes signature based on the IMMPORT algorithm showed that the AUC values of 3-year and 5-year OS were 0.663 and 0.713, respectively. Although the other two algorithms do not calculate the 1-year AUC, our model has higher 3-year and 5-year AUC than other models (Table 1). We can preliminarily conclude that the immune-related gene signature we obtained through the ESTIMATE algorithm has higher accuracy.

**Table 1:** Comparison of our immune-related gene signature using the ESTIMATE algorithm with immune-related gene signature using other methods.

**Immune-related gene signature**	**AUC values**		
	**1-Year**	**3-Year**	**5-Year**
**Entire TCGA cohort**			
Using ESTIMATE (Our model)	0.795	0.757	0.728
Using CIBERSORT (PMID 31689990)	-	0.676	0.661
Using IMMPORT (PMID 32375704)	-	0.663	0.713

## DISCUSSION

Because of the heterogeneity and complexity of tumor immune microenvironments, only some microsatellite instable colon cancer patients benefit from immunotherapy. It remains critical to construct an effective model for accurately predicting the immunotherapy prognosis of colon cancer patients. To our knowledge, this is the first study to apply the ESTIMATE algorithm to identify the immune-related prognostic signature of colon cancer. We validated the independent prognostic effects of IRG signature, the robustness of IRG signature in the external cohort, and the association of IRG signature with somatic cell mutations and immune cell infiltration. On this basis, we explored the association between risk score and immune checkpoint expression and identified the optimal beneficiaries of immune checkpoint inhibitors in colon cancer for the first time. Compared with models based on other methods, the immune-related signature we derived using the ESTIMATE algorithm has higher accuracy. Four immune-related genes were verified by immunohistochemistry in the HPA database, while CCL22 and TRPM5 need further experimental verification.

The ESTIMATE algorithm was used to calculate immune and stromal scores. The results showed that a higher immune score was associated with better overall survival, indicating that TME was related to the prognosis of colon cancer. Similar associations were found in adrenocortical carcinoma, endometrial carcinoma, hepatocellular carcinoma, non-small cell lung cancer, and melanoma [18–22]. Furthermore, we observed that an immune score of M1 and stage IV was significantly lower than that of M0 and stage I. Immune or stromal scores were calculated based on a comprehensive score of all genes in the tissue [14]. Although immune score was related to prognosis, not all immune-specific genes were prognostic factors. Similarly, although stromal score was not associated with prognosis, stromal-specific genes are not necessarily unrelated to prognosis [23]. Since the intersect genes are the most comprehensive and conservative, we took the intersection of immunity and stroma to obtain DEGs related to both immunity and stroma. GO analysis showed that 844 DEGs are involved in immune-related biological processes such as immune response, inflammatory response, cell adhesion and innate immune response. KEGG analysis revealed that DEGs enriched in immune-related pathways including cytokine-cytokine receptor interaction, phagosome, and chemokine signaling pathways. The activation of cytokine-cytokine receptor interaction could promote intestinal tumorigenesis [24]. The main role of the chemokine signaling pathway is to regulate immune cell recruitment during inflammation and defense against external pathogens [25, 26]. New evidence has indicated that chemokines are a key component of cancer progression [27], and the functions of different chemokines are complex and diverse in the tumor microenvironment [28]. CXCL2 significantly promotes tumor migration and invasion [29], whereas overexpression of CXCL2 inhibits tumor growth and promotes apoptosis [30]. CXCL11 promotes tumor cell proliferation and invasion by inducing macrophage infiltration, which leads to poor prognosis of colon cancer [31, 32]. Conversely, another study reports that CXCL11 and CXCL10 have synergistic antitumor effects [33]. Indeed, more and more studies are showing that the stromal differential expression of chemokines is related to the prognosis of cancer [34, 35]. These enriched functions and pathways could provide a reference for fundamental research on the molecular mechanisms of DEGs.

The six genes that composed the risk score could be considered potential therapeutic targets. Among these, AXIN2, CCL22, and CLEC10A are the protective factors in the model. Axis Inhibition Protein 2 (AXIN2) is a protein coding gene. It presumably plays an important role in the regulation of the stability of beta-catenin in the WNT signaling pathway. Intestinal tumor suppressor CDX2 activated AXIN2 promoter activities via intestinal cell-specific enhancer elements to inhibit WNT signaling pathways and prevent tumor invasion and migration [36]. Therefore, up-regulation of AXIN2 could restore immune cell infiltration and enhance immunotherapy through inhibitory WNT/β-catenin pathways [37]. C-C Motif Chemokine Ligand 22 (CCL22), generated by intestinal epithelial cells, produces anti-inflammatory cytokines (IL-4, IL-10) through chemotactic T cell capacity, thereby regulating physiological mucosal inflammation [38, 39]. CCL22 knockout mice exhibited impaired immune response and increased mortality, which was associated with reduced macrophage recruitment [40, 41]. C-type lectin receptor family member 10A (CLEC10A) is an endocytosis receptor on antigen-presenting cells that plays an important role in dendritic cell maturation and initiation of immune response [42]. As an immunotherapy target, [43, 44] CLEC10A has proven to be an effective tool for activating the immune response in ovarian cancer [45] although its function in colon cancer has not been explored.

High expression of CRIP2, RUNX3, and TRPM5 was significantly associated with an inferior prognosis. Cysteine Rich Protein 2 (Cysteine Rich Protein 2) may promote apoptosis of esophageal squamous cell carcinoma cells. However, the functional role of CRIP2 and its involvement in colon tumorigenesis are still unknown. Runt related (RUNX) family of transcription factors, including runx1, runx2, and runx3, have been proposed to be key lineage-specific developmental regulators that are associated with multiple cancers. RUNX Family Transcription Factor 3 (RUNX3) is a downstream target of the TGF-β pathway, which is a tumor suppressor in pathway. RUNX3 has been described as a tumor suppressor gastric cancer and lung cancer [46, 47]. Paradoxically, RUNX3 has also been reported to play a carcinogenic role in basal cell carcinoma, head and neck squamous cell carcinoma, and ovarian cancer, which could promote tumor frequency and probability [48–50]. In any case, RUNX3 is a key target for tumor therapy, and its specific molecular mechanisms need to be explored in the future. It has been reported that forced expression of Transient Receptor Potential Cation Channel Subfamily M Member 5 (TRPM5) increases the rate of acidic PHE-induced matrix metalloproteinase-9 expression and experimental lung metastasis. It has also been suggested that high expression of TRPM4, from the same family, is associated with aggressive tumor features and metastasis in colorectal cancer [51]. Five of these prognostic genes are specifically involved in immune-related processes (AXIN2, CCL22, CLEC10A, RUNX3, and TRPM5). Therefore, these five genes could be considered potential immunotherapy targets.

To verify the accuracy of this information, we analyzed the six immune-related genes together with the risk scores in the GEO cohort. We found that protective genes were negatively correlated with risk scores, while risk genes were positively correlated with risk scores, which was consistent with the TCGA data set. We also demonstrate that the proteins encoded by the six immune-related genes were expressed at different degrees in tumor tissues and normal tissues. Images of IHC staining of AXIN2, CLEC10A, CRIP2, and RUNX3 verified the accuracy of the results, while IHC staining of the CCL22 and TRPM5 genes needs to be further verified.

Somatic mutation analysis showed that our IRG signature was positively correlated with the frequency of somatic cell mutation. Since high somatic mutation means microsatellite instability-high, which has a higher immunotherapy response, the immunotherapy effect of the IRG high-risk group with high somatic mutation may be better. Furthermore, immune cell infiltration analysis showed high proportions of resting NK cells, regulatory T cells in the high-risk group. In contrast, low-risk group tissues had higher fractions of CD8 T cells, plasma cells, memory-activated CD4 T cells, resting dendritic cells, and activated dendritic cells. Research increasingly demonstrates that immune checkpoints are important influencing factors in tumor prognosis. Our results showed a significant positive correlation between risk scores and the expression of four key immune checkpoints (PD-1, PD-L1, LAG3, and VSIR). Previous evidence has suggested that the upregulation of PD-L1 on dendritic cells is related to the high expression of PD-1 on T cells, which means that dendritic cells present tumor antigens to T cells and inhibit anti-tumor responses [52]. Inhibition of PD-L1, LAG3, and CTLA4 could increase CD8 T cells and CD4 T cells and reduce Tregs, thereby enhancing anti-tumor response [53–55]. The above studies are consistent with our conclusion, indicating that the poor prognosis in the high-risk group may be caused by a high expression of immune checkpoints and immunosuppressive microenvironments that promote the development of colon cancer. Therefore, immunosuppressive agents may be more effective for high-risk patients.

We found correlations between IRG signature and somatic mutation, immune cell infiltration, and immunosuppression checkpoints, suggesting that the optimal beneficiaries of immunotherapy are high-risk groups. We also validated the prognostic value of IRG characteristics in different subgroups and external cohorts. A multivariable Cox regression analysis combining clinical features with IRG risk scores showed that age, pathological stage, and risk score were significantly correlated with prognosis. Therefore, three independent prognostic factors were used to construct a nomogram. The AUC values for 1-, 3-, and 5-years on the nomogram were higher than those on the gene risk score model, which indicates that the nomogram has better accuracy and sensitivity than single factor prediction models.

Several recent studies have been committed to finding the immune signature of colon cancer prognosis. Pan et al. (2019) demonstrated that LAYN can be used as a prognostic biomarker for determining prognosis and immune infiltration in colon cancer using the TIMER site. Nevertheless, the accuracy and information of single immune-related biomarkers was lower than that of our multi-gene comprehensive model. Zhao et al. (2019) filtered and selected the immune-related genes using the criterion of a CIBERSORT *P*-value < 0.05. Chen et al. (2020) obtained immune genes from the IMMPORT database. The AUC of the model constructed by these methods is lower than that of the ESTIMATE algorithm adopted in this study.

The present study differs from previous reports about colon cancer prognosis and has its own advantages. First, the ESTIMATE algorithm was applied to study genes characteristics in colon cancer microenvironment for the first time, and these genes affect the development of colon cancer and hence contribute to patients’ overall survival. Second, since colon cancer is a typical microsatellite-instable tumor, it is necessary to study its TMB and immune cell infiltration to establish the efficacy of immunotherapy. This study was the first to comprehensively analyze the relationship between IRG prognostic signature and TMB as well as immune cell infiltration, providing a new perspective on the predictive function of IRG signature in immunotherapy. Third, this study is the first to explore the correlation between the IRG signature and expression of immune checkpoint inhibitors and identify the optimal beneficiaries in clinical immune checkpoint inhibitor therapy. Our results may provide additional evidence for exploring the complex interactions between tumor, immunotherapy, and tumor environment in colon cancer.

Nevertheless, our current research still has a few limitations. First, the main source of clinical characteristics for our dataset was the TCGA database. The majority of patients were from North America, and thus, we should extend our findings to other geographical and ethnic groups with great caution. Second, our study provides evidence that six novel immune-related genes are significantly related to the prognosis of colon cancer patients, but they were analyzed only through data mining merely. The function and mechanism of these genes depend on further experimental studies to elucidate. Thirdly, although we adjusted for the demographics and clinical characteristics as much as possible, information on other potentially variables (e.g., smoking, BMI) was not included in the present study. Finally, our retrospective study could lead to reporting bias. Thus, the results of new IRG signature need to be further validated in prospective studies.

## CONCLUSIONS

In summary, we have demonstrated the effectiveness of the ESTIMATE algorithm applied for screening immune-related genes of colon cancer. IRG signature of AXIN2, CCL22, CLEC10A, CRIP2, RUNX3, and TRPM5 is a reliable prognostic predictor for colon cancer patients. We also found that the IRG signature is related to immune cell infiltration and expression of immune checkpoints, which further identified the optimal beneficiaries in clinical immune checkpoint inhibitor therapy. This new IRG signature provides a new theoretical basis for the prognosis assessment of colon cancer patients, and is expected to be further applied in future clinical practice.

## MATERIALS AND METHODS

### Data download and preparation

Fragments per kilobase million (FPKM) data on RNA-Seq and corresponding clinical characteristics of colon cancer were downloaded from The Cancer Genome Atlas (TCGA, https://portal.gdc.cancer.gov/, up to June 06, 2020) using the Genomic Data Commons (GDC) tool. The original data included 482 tumor tissues and 42 adjacent tissues. FPKM data was translated into transcripts per million (TPM) expression data. Based on the requirement of data integrality, patients that met the following criteria were excluded from the subsequent analysis: (1) repeated patient records; (2) missing follow-up time, T/N/M staging, or pathological stage information; (3) follow-up time was 0 days. Finally, our study identified 415 colon cancer samples as analytical datasets.

This study selected the GSE39582 dataset from the Gene Expression Omnibus (GEO, http://www.ncbi.nlm.nih.gov/geo/) for external validation. The GSE39582 dataset was based on a GPL570 platform (Affymetrix Human Genome U133 Plus 2.0 Array) and included 558 colon cancer patients and 19 normal subjects. Our study eventually included 519 patients after deleting 38 samples that had a follow-up time of 0. The raw data were processed by RMA background correction, log2 transformation and normalization using an “affy” package. All data were from publicly available databases, and there were no ethical issues involved.

### Identification of immune/stromal related-DEGs and functional enrichment analysis

The ESTIMATE algorithm (https://sourceforge.net/projects/estimateproject/) was used to calculate immune scores and stromal scores [14]. For samples that had a normal distribution, an independent *t*-test was performed to compare immune and stromal scores between two groups of clinical characteristics; one-way analysis of variance was used for comparison among three or more groups. Otherwise, Wilcoxon and Kruskal-Wallis analysis were conducted for two and three or more groups, respectively. We used the "maxstat" statistic from the "survminer" package to identify the optimal cut-off for continuous variables [56]. Immune and stromal scores were classified into high and low score groups, respectively, according to the optimal cut-off values. Overall survival (OS) was estimated by Kaplan-Meier, and a log-rank test was employed to compare survival differences between the two groups.

Differential expression analysis of high and low score groups was performed with a "limma" package [57]. The *P*-value was adjusted with the false discovery rate (FDR) [58]. The up- and down-regulated immune and stromal genes were obtained based on the criteria of fold change ≥1.5 and adjusted FDR <0.05. The intersection between immune-related differentially expressed genes (DEGs) and stromal-related DEGs was identified by using the VENNY website (https://bioinfogp.cnb.csic.es/tools/venny/). Heat maps and volcano plots of DEGs were constructed using the “pheatmap” and “ggplot2” packages.

The Gene Ontology (GO) and Kyoto Encyclopedia of Genes and Genomes (KEGG) pathway enrichment analysis of DEGs were implemented by the Database for Annotation, Visualization, and Integrated Discovery (DAVID) (https://david-d.ncifcrf.gov/) [59–61]. GO analysis included three main parts: biological process (BP), cellular component (CC), and molecular function (MF). We selected the top ten gene ontology terms in three parts to draw the histogram. The top fifteen KEGG analysis terms were exhibited in the bubble chart (where an adjusted *P*-value <0.05 was considered statistically significant).

### Construction, validation, and subgroup analysis of prognostic risk score model

First, a univariate analysis of differential genes was performed to screen out significant genes. Subsequently, the least absolute shrinkage and selection operator (LASSO) Cox regression model was utilized for further screening of prognostic genes to avoid overfitting the model. Finally, a Cox proportional hazard regression was used to determine the optimal prognostic genes of the model [62]. The formula of the gene signature is as follows: Risk score = Ʃ (βi * Expi) (i = the number of prognostic genes, βi represents the gene coefficient, and Expi represents gene expression). Colon cancer patients were divided into high-risk and low-risk groups based on the median risk score. The “survival”, “glmnet”, “survminer”, and “forestplot” packages were used to conduct the above analysis.

The risk score model was validated by 519 colon cancer patients from the GSE39582 dataset. The risk scores of the GSE39582 dataset were calculated based on the above formula. The GSE39582 dataset was also divided into high-risk and low-risk groups based on the median risk score. The performance of the risk score model in the training set and validation set was assessed based on a time-dependent receiver operating characteristic curve (ROC) analysis realized by “timeROC” [63]. An area under time-dependent receiver operating characteristic curve (AUC) greater than 0.75 indicated clearly useful discrimination, while an AUC of 0.60 to 0.75 indicated possibly useful discrimination.

To explore the association between the IRG signature and clinical characteristics, box plots were used to show risk scores for differences in age groups, gender, race, pathological type, tumor location, and tumor stage. A *t*-test was used to compare whether there were significant differences in risk scores between different clinical features. A Kaplan-Meier method and log-rank test were used to compare the survival curves of different clinical features. To determine that the IRG signature was significant for survival prediction between different clinical characteristics, a survival analysis was performed on each subgroup.

### Expression profile and immunohistochemistry (IHC) validation of immune-related genes

Scatter plots were used to show the distribution of gene expression profiles and risk scores. Pearson correlation coefficients were used to indicate the correlation between gene expression profiles and risk scores in the GEO cohort. Images of immunohistochemistry (IHC) staining of the selected prognosis-related genes in normal tissue and colon cancer tissue were retrieved from the Human Protein Atlas (HPA) database (http://www.proteinatlas.org).

### Correlations between somatic mutation, immune cell infiltration, immune checkpoint expression, and IRG signature

CIBERSORT is a deconvolution algorithm for immune cell subtype expression based on linear support vector regression [17]. LM22 provides the annotated gene expression signatures for 22 immune cell subtypes, including plasma cells, myeloid subsets, seven T cell types, naive and memory B cells, and natural killer (NK) cells. Compared with traditional immunohistochemistry and flow cytometry methods, the CIBERSORT algorithm can comprehensively, quickly, and accurately infer the relative proportion of the 22 invasive immune cells in tumors [17]. Immune cell fractions from high- and low-risk groups were analyzed using the CIBERSORT algorithm in our research. Immune checkpoint expression was used to predict the immunotherapeutic benefits of various malignancies. Therefore, we assessed the association between the expression of five common immune checkpoints—PD-1, CTLA-4, lymphocyte-activation gene 3 (LAG3), programmed cell death 1(PD-1), and V-Set immunoregulatory receptor (VSIR), with the IRG signature. A circular plot showed the correlation between the risk score and the expression of the immune checkpoint, which was implemented with the "circlize" package. A box plot showed the differences in immune checkpoint expression between high- and low-risk groups.

### Verification that IRG risk score is an independent prognostic factor and construction of a nomogram

To assess the independent prognostic effect of the gene risk model, a Cox proportional hazards regression was used to perform univariate and multivariate analysis on all possible prognostic factors. Histological classification included adenocarcinoma and non-adenocarcinoma. Tumor location referred to left-side (descending colon, sigmoid colon, splenic flexure of colon), right-side (ascending colon, cecum, hepatic flexure of colon, transverse colon), or unknown [64]. Age, sex, pathological stage, and T/N/M stage were considered categorical variables.

The nomogram was applied as a visual tool for predicting survival probability. All significant prognostic factors selected by multivariate Cox regression analysis were used to construct a nomogram to obtain a total score, thereby predicting 1-, 3-, and 5-year survival probabilities. Subsequently, time-dependent ROC and calibration were used to assess the performance of the nomogram. If probabilities approach the 45-degree angle line in a calibration plot, it means there is strong agreement between the nomogram prediction and the actual observations.

## Supplementary Material

Supplementary Figures

Supplementary Tables
