# Supplementary material for: Identification and validation of an immune-related gene signature predictive of overall survival in colon cancer
Source: Aging (Albany NY). 2020 Dec 19;12(24):26095–120. doi: 10.18632/aging.202317 (PMC7803520; doi:10.18632/aging.202317)
Supplement: Supplementary Figures [file aging-12-202317-s001.pdf]

SUPPLEMENTARY FIGURES

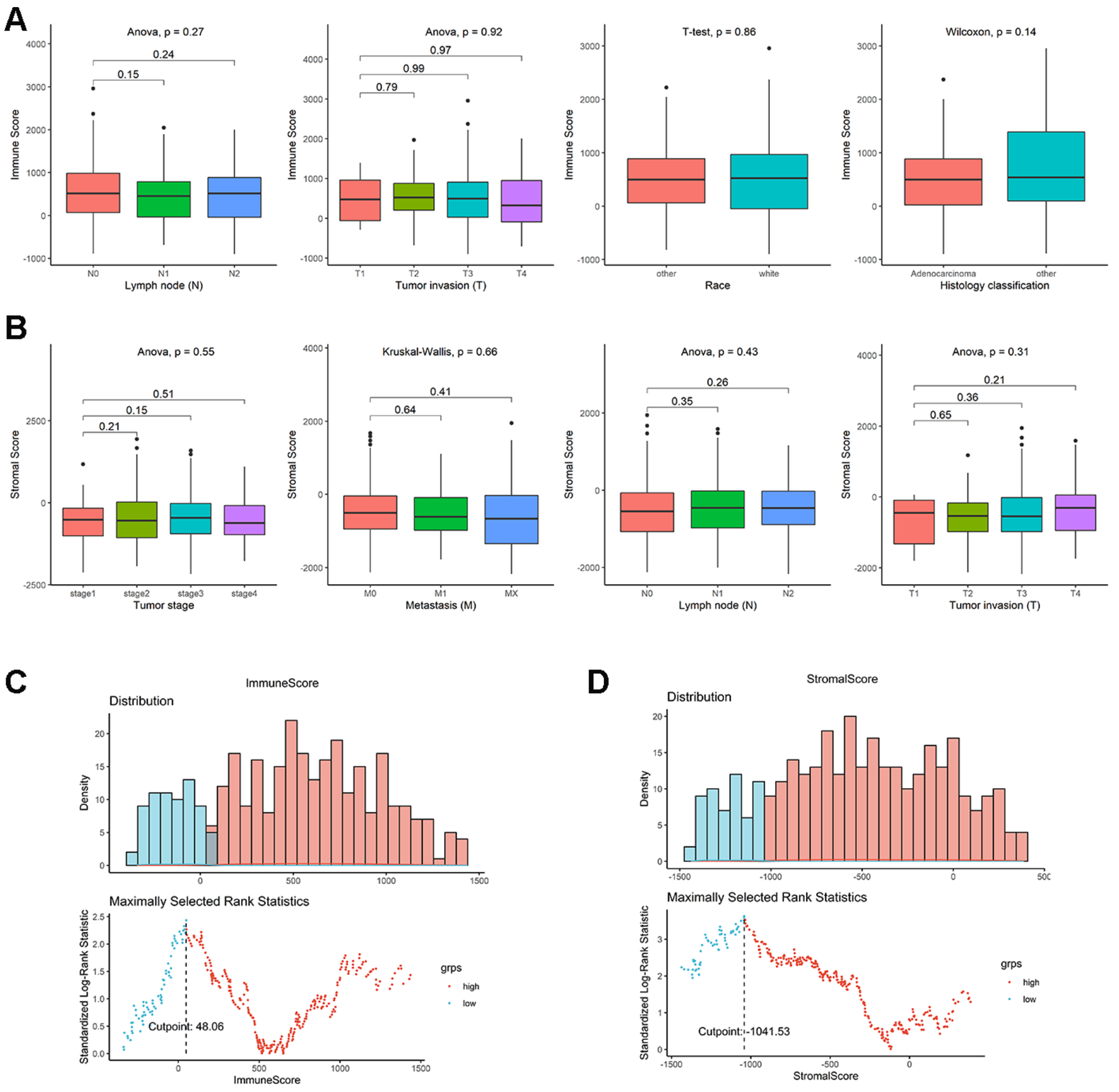

Supplementary Figure 1. Cutoff values for high and low immune/stromal scores in TCGA. (**A**) No significant differences in the distribution of immune scores among different lymph node, tumor invasion, race, and histology classification groups. (**B**) No significant differences in the distribution of stromal scores among different tumor stage, metastasis, lymph node, and tumor invasion groups. (**C**) An illustration of optimal cutoff identification for immune scores. The scatter plot shows the standardized log-rank statistic value for each corresponding expression cutoff. The optimal cutoff with the maximum selected log-rank statistic is marked with a vertical dashed line. The histogram shows the density distribution for low and high immune score groups divided by the optimal cutoff. (**D**) An illustration of optimal cutoff identification for stromal scores.

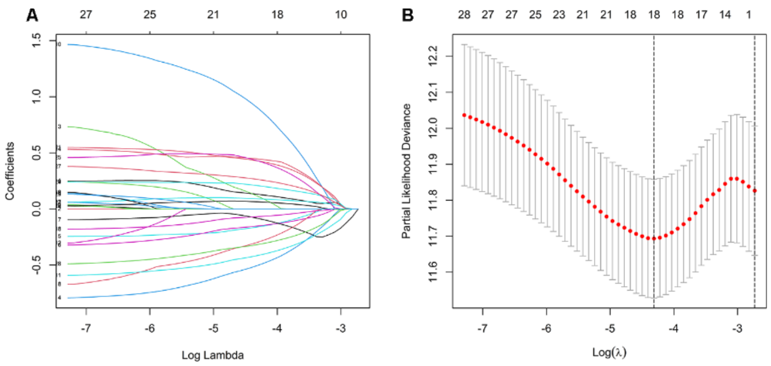

Supplementary Figure 2. Eighteen DEGs were identified by a LASSO regression analysis. (**A**) LASSO coefficient profiles of the TME-related prognostic DEGs. (**B**) Partial likelihood deviance of different numbers of variables revealed by the LASSO regression model**.**

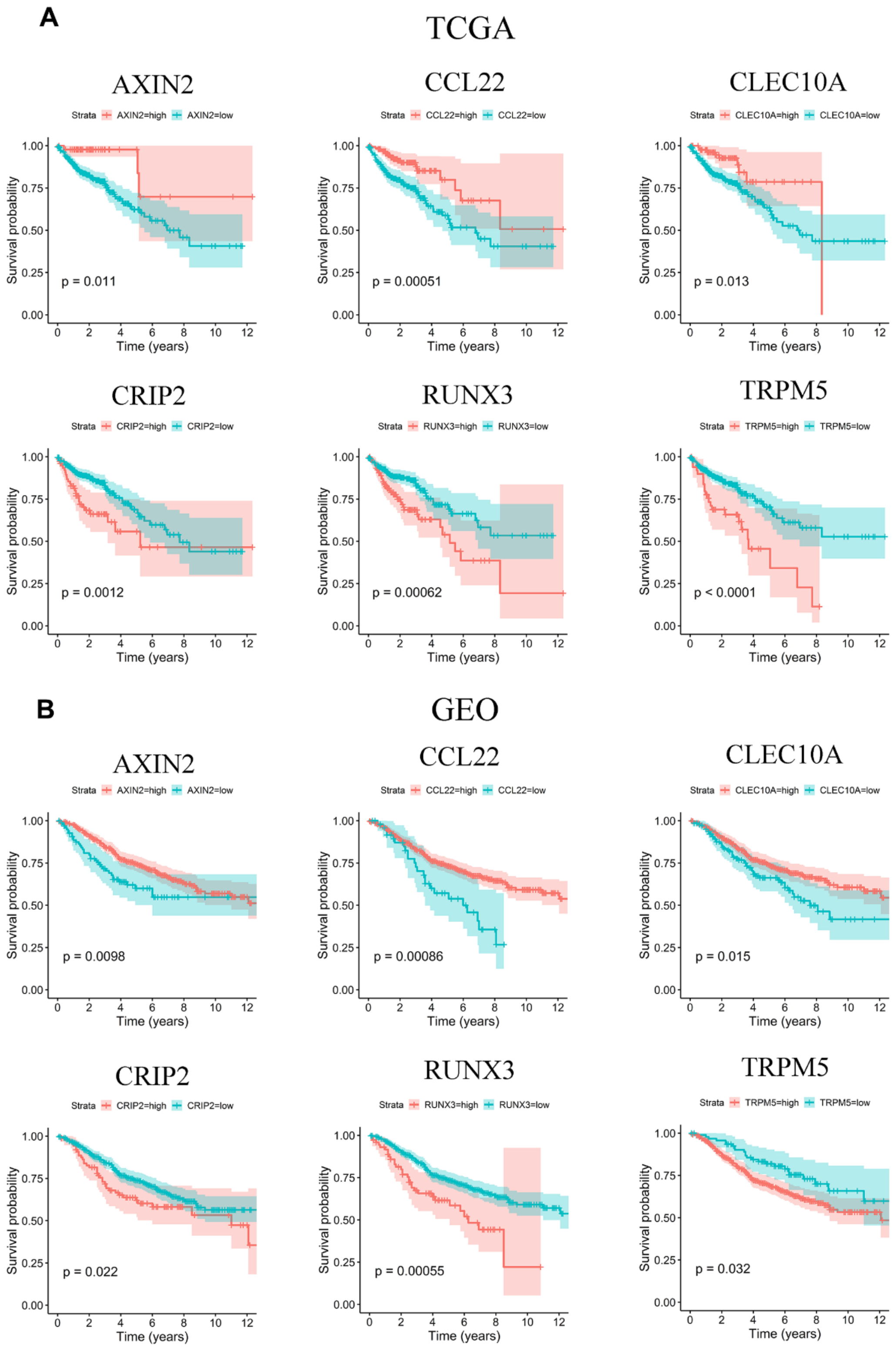

Supplementary Figure 3. Kaplan-Meier survival curves and log-rank tests of six prognosis genes in TCGA and GEO. (**A**) Kaplan-Meier plots of overall survival for patients from TCGA, grouped by expression of six prognosis genes, AXIN2, CCL22, CLEC10A, CRIP2, RUNX3, and TRPM5. (**B**) Kaplan-Meier plots of overall survival for patients from GEO, grouped by expression of six prognosis genes, AXIN2, CCL22, CLEC10A, CRIP2, RUNX3, and TRPM5.

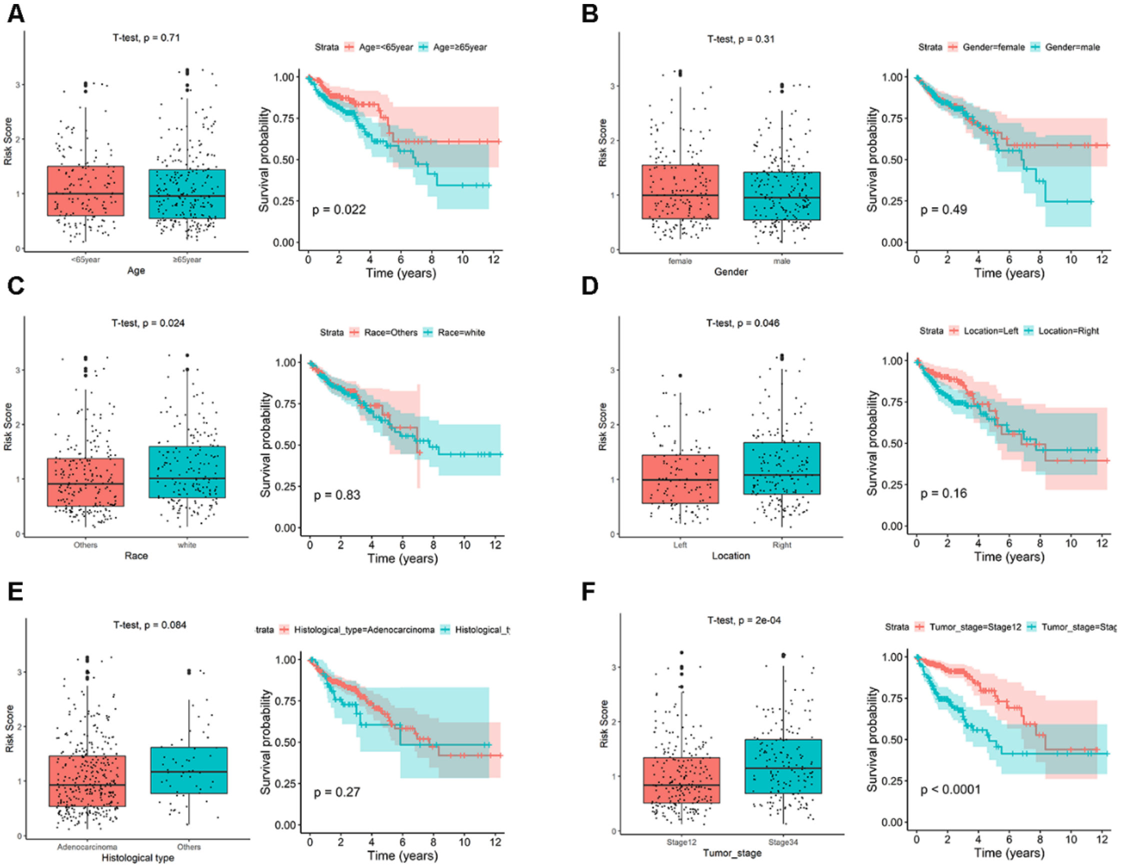

Supplementary Figure 4. Boxplots and survival analysis for correlation test between clinical characteristics and IRG signature in the TCGA. (**A**) Age <65 years and age ≥65 years. (**B**) Female and Male. (**C**) White race and other race. (**D**) Left site and right site. (**E**) Adenocarcinoma and other histological type. (**F**) Stage I/II and stage III/IV.

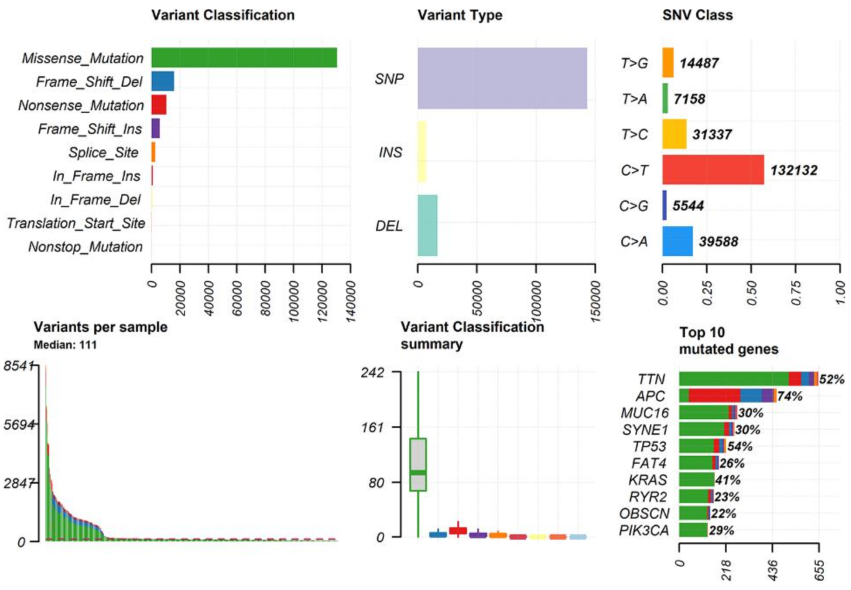

Supplementary Figure 5. Tumor mutation burden and mutation distribution of all colon cancer patients.

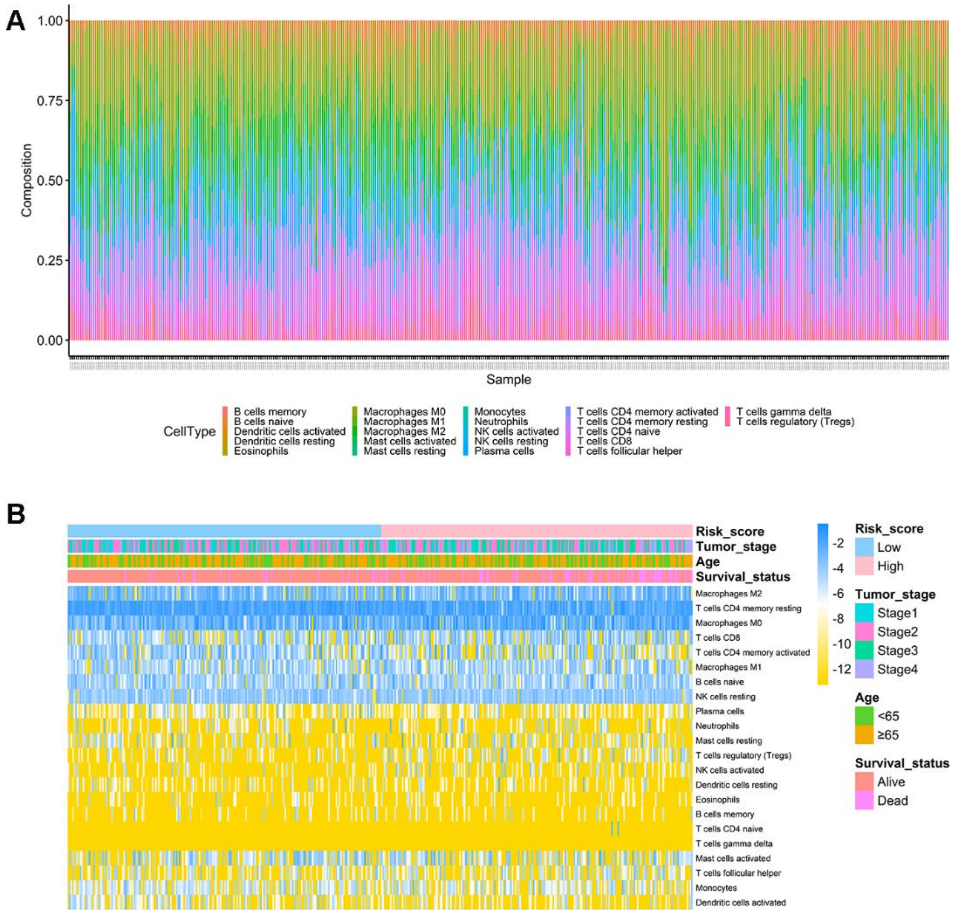

Supplementary Figure 6. Immune infiltrations of high-risk and low-risk groups in TCGA. (**A**) Relative proportion of immune infiltration in high-risk and low-risk groups. (**B**) Heat map of 22 immune cell proportions in high- and low-risk groups.
