# Supplementary material for: Identification and validation of an immune-related gene signature predictive of overall survival in colon cancer
Source: Aging (Albany NY). 2020 Dec 19;12(24):26095–120. doi: 10.18632/aging.202317 (PMC7803520; doi:10.18632/aging.202317)
Supplement: Supplementary Tables [file aging-12-202317-s002.pdf]

SUPPLEMENTARY TABLES

Supplementary Table 1. The clinical characteristics in TCGA [n (%)].

Variables	TCGA cohort (N=415)	GEO cohort (N=519)
**Age**		
<65	157(37.8)	192(37.0)
≥65	258(62.2)	327(63.0)
**Gender**		
male	221(53.3)	233(44.9)
female	194(46.8)	286(55.1)
**Race**		
White	192(46.3)	
Others	223(53.7)	
**Tumor invasion**		
T1	9(2.2)	11(2.1)
T2	72(17.4)	43(8.3)
T3	285(68.7)	337(64.9)
T4	49(11.8)	105(20.2)
Unknown		23(4.4)
**Metastasis**		
M0	317(76.4)	468(90.2)
M1	60(14.5)	30(5.8)
Unknown	38(9.2)	21(4.0)
**Lymph node**		
N0	245(59.0)	281(54.1)
N1	98(23.6)	124(23.9)
N2	72(17.4)	85(16.4)
N3		5(1.0)
Unknown		24(4.6)
**Pathological stage**		
Stage I	73(17.6)	36(6.9)
Stage II	164(39.5)	253(48.7)
Stage III	118(28.4)	200(38.6)
Stage IV	60(14.5)	30(5.8)
**Tumor location**		
Left	125(30.1)	310(59.7)
Right	186(44.8)	209(40.3)
Unknown	104(25.1)	
**Histological classification**		
Adenocarcinoma	351(84.6)	
Others	64(15.4)	

Supplementary Table 2. Numbers of mutations and numbers of patients with mutations in high- and low-risk groups.

Pathway	No. of mutations		No. of patients with mutations(%)		
	**high-risk**	**low-risk**	**high-risk**	**low-risk**	***P***
RTK-RAS	81	78	151(85.3%)	135(75.4%)	**0.019**
WNT	64	61	164(92.7%)	161(89.9%)	0.364
NOTCH	62	61	103(58.2%)	81(45.3%)	**0.015**
Hippo	37	31	104(58.8%)	96(53.6%)	0.330
PI3K	28	23	96(54.2%)	90(50.3%)	0.455
MYC	13	10	39(22%)	18(10.1%)	**0.002**
Cell-Cycle	10	6	30(16.9%)	8(4.5%)	**<0.001**
TGF-Beta	7	7	70(39.5%)	46(25.7%)	**0.005**
TP53	6	6	115(65%)	113(63.1%)	0.717
NRF2	3	2	13(7.3%)	6(3.4%)	0.094
